# A method to construct a points system to predict cardiovascular disease considering repeated measures of risk factors

**DOI:** 10.7717/peerj.1673

**Published:** 2016-02-15

**Authors:** Antonio Palazón-Bru, Julio Antonio Carbayo-Herencia, Maria Isabel Vigo, Vicente Francisco Gil-Guillén

**Affiliations:** 1 Department of Clinical Medicine, Miguel Hernández University, San Juan de Alicante, Alicante, Spain; 2 Chair of Cardiovascular Risk, San Antonio Catholic University, Murcia, Murcia, Spain; 3 Department of Applied Mathematics, University of Alicante, San Vicente del Raspeig, Alicante, Spain

**Keywords:** Cardiovascular diseases, Cardiovascular models, Risk factors, Cohort studies

## Abstract

Current predictive models for cardiovascular disease based on points systems use the baseline situation of the risk factors as independent variables. These models do not take into account the variability of the risk factors over time. Predictive models for other types of disease also exist that do consider the temporal variability of a single biological marker in addition to the baseline variables. However, due to their complexity these other models are not used in daily clinical practice. Bearing in mind the clinical relevance of these issues and that cardiovascular diseases are the leading cause of death worldwide we show the properties and viability of a new methodological alternative for constructing cardiovascular risk scores to make predictions of cardiovascular disease with repeated measures of the risk factors and retaining the simplicity of the points systems so often used in clinical practice (construction, statistical validation by simulation and explanation of potential utilization). We have also applied the system clinically upon a set of simulated data solely to help readers understand the procedure constructed.

## Introduction

Given that cardiovascular diseases (CVD) are one of the main causes of death in the world (World Health Organization, 2014), prediction models are interesting in order to determine those risk factors that can be acted on to reduce the probability of CVD (Molinero, 2003). The simplest model to make predictions about a dichotomous event, such as CVD, is logistic regression (Hosmer & Lemeshow, 2000). This model produces an equation which, once the values for the various risk factors are known, can be used to evaluate the likelihood of the appearance of disease. However, this sort of model fails to consider exposure time. This is precisely what is done in survival models, which analyse the time of occurrence of a particular event. Although the best known of these models is Cox (Hosmer, Lemeshow & May 2008), it is not the only alternative available. There exist other possible methods to analyse survival, called parametric models as they assume a concrete type of distribution, such as the Weibull model, used in the SCORE project (Conroy et al., 2003). Indeed, the Framingham study used both logistic regression models and survival models (parametric and non-parametric) (National Heart, Lung, and Blood Institute, 2015).

In conjunction with the Framingham and SCORE predictive models, others have been developed that are also used in clinical practice, though to a lesser extent, such as the Reynolds risk score and the WHO/ISH score (Cooney, Dudina & Graham, 2009). Common to all these is the making of predictions about CVD over a 10-year period, though they consider different outcomes (morbidity and mortality with coronary heart disorders, mortality from coronary heart disorders, cardiovascular morbidity and mortality, or just cardiovascular mortality) and use different mathematical models (Cox and Weibull). These models enable physicians to make long-term decisions for their patients. In addition, the clinical practice guidelines recommend using these predictive models to stratify the cardiovascular risk of patients. For example, in Europe, the European Guidelines on cardiovascular disease prevention in clinical practice indicate “A risk estimation system such as SCORE can assist in making logical management decisions, and may help to avoid both under-and overtreatment” (Perk et al., 2012). In other words, clinicians follow the guidelines to improve the decision-making process in order to prevent CVD, and it is these very guidelines that indicate the use of these predictive models. Accordingly, these models are very relevant in daily clinical practice.

Given the complexity of these mathematical models an algorithm is used to enable the clinician to understand them more easily, though precision is lost in the estimation of the probability of CVD (Sullivan, Massaro & D’Agostino, 2004). To do this, the mathematical models have been transformed into coloured risk tables that can be used systematically in clinical practice. However, these tables are based on models that manage clinical variables in the baseline situation of the patient (Conroy et al., 2003; National Heart, Lung, and Blood Institute, 2015), and do not therefore take into account the variability of the variables over time, as the biological parameters are being considered constant over the follow-up period when in fact they vary greatly and the physician can intervene using drugs to either reduce or increase their value (National Cholesterol Education Program, 2002; American Diabetes Association, 2014; James et al., 2014; Stone et al., 2014).

Predictive models for survival in other diseases do consider the temporal variability of a single biological marker (as well as the baseline variables). These are known as Joint Models for Longitudinal and Time-to-Event Data and comprise two parts: (1) A mixed linear model to determine the path of a longitudinal parameter; and (2) A survival model relating the baseline variables and the longitudinal parameter with the appearance of an event. These models can be used to make more precise predictions about the development of a disease (Rizopoulos, 2012). However, due to their complexity they are not used in general clinical practice. In addition, joint modelling when the survival part is formed by a linear function with multiple longitudinal parameters (usual modelling in traditional survival analysis in the health sciences) has only been examined theoretically and currently remains a complete computational challenge. This has resulted in the development of algorithms to make predictions, as in the univariate case (Rizopoulos, 2011).

Here we aim to show the viability and properties of a new methodological alternative for constructing cardiovascular risk scores (construction, statistical validation by simulation and potential utilization with the new theoretical model) dealing with the temporal variability of CVD risk factors. We also apply the model using a set of simulated data, with the sole purpose of helping readers understand how to apply it to a real data set with repeated measures of cardiovascular risk factors. In other words, the example given using simulated data is only to show how to apply the method proposed with a real data set having the characteristics given in this work. Thus, the scoring system given here has no value in clinical practice; what is of value is the way the system is constructed.

## Materials and Methods

The basic models used to develop the new method were the Cox model with time-dependent variables, points system in the Framingham Heart Study, Joint Models for Longitudinal and Time-to-Event Data, and predictions of the longitudinal biomarkers using these Joint Models.

### Cox model with time-dependent variables

Let *T* be a non-negative random variable denoting the observed failure time, which is the minimum value of the true event time *T*^∗^ and the censoring time *C* (non-informative right censoring). In other words, *T* = min(*T*^∗^, *C*). In addition, we define *δ* as the event indicator, which takes the value 1 if *T*^∗^ ≤ *C* and 0 otherwise. On the other hand, let *W* be the vector of baseline covariates and *Y*(*t*) the vector of time-dependent covariates, assuming a defined value for *t* ≥ 0. With these data, the Cox model with time-dependent variables takes the following form (risk function):

\begin{eqnarray*}h \left( t{|}w,y \left( t \right) \right) ={h}_{0} \left( t \right) \exp \left\{ {\gamma }^{T}w+{\alpha }^{T}y \left( t \right) \right\} , \end{eqnarray*}

where *h*_0_(*t*) is the baseline risk function, and *γ* and *α* are the vectors of the regression coefficients for the baseline and time-dependent covariates, respectively (Andersen & Gill, 1982).

The estimation of the model parameters is based on the partial likelihood function (Andersen & Gill, 1982). On the other hand, we have to corroborate whether the functional form of the covariates in the model is linear. This should be performed using graphical methods (Martingale residuals against the covariate of interest). Finally, we have to assess whether the model fits the data well, through the analysis of the Cox-Snell residuals (graphical test).

The classical Cox regression model (with no time-varying covariates), deletes *α* and **y**(*t*) from the above expression. Furthermore, the model has to verify the following condition (proportional hazard assumption):

\begin{eqnarray*}\log \left( \frac{h \left( t{|}w \right) }{{h}_{0}(t)} \right) ={\gamma }^{T}w. \end{eqnarray*}

### Points system in the Framingham Heart Study

We summarize the steps of the method developed by the Framingham Heart Study to adapt a Cox regression model with *p* covariates to risk charts (Sullivan, Massaro & D’Agostino, 2004):

(1) Estimate the parameters of the model: $\hat {\gamma }$.
(2) Organize the risk factors into categories and determine reference values:
  (a) Continuous risk factor (e.g., age): set up contiguous classes and determine reference values for each. Example for age: 18–30 [24], 30–39 [34.5], 40–49 [44.5], 50–59 [54.5], 60–69 [64.5] and ≥70 years [74.5]. In brackets is the reference value. The Framingham Heart Study researchers recommend mid-points as acceptable reference values, and for the first and last class the mean between the extreme value and 1st (first class) or 99th percentiles (last class).
  (b) Binary risk factors (e.g., gender, 0 for female and 1 for male): the reference value is again either 0 or 1.
    Let *W*_*ij*_ denote the reference value for the category *j* and the risk factor *i*, where *i* = 1, …, *p* and *j* = 1, …, *c*_*i*_ (total number of categories for the risk factor *i*).
(3) Determine the referent risk factor profile: the base category will have 0 points in the scoring system and it will be denoted as *W*_*iREF*_, *i* = 1, …*p*.
(4) Determine how far each category is from the base category in regression units: calculate ${\hat {\gamma }}_{i}\cdot \left( {W}_{ij}-{W}_{iREF} \right) $, *i* = 1, …, *p* and *j* = 1, …, *c*_*i*_.
(5) Set the fixed multiplier or constant *B*: the number of regression units equivalent to 1 point in the points system. The Framingham Heart Study generally uses the increase in risk associated with a 5-year increase in age.
(6) Determine the number of points for each of the categories of each risk factor: the closest integer number to ${\hat {\gamma }}_{i}\cdot \left( {W}_{ij}-{W}_{iREF} \right) {}B$.
(7) Determine risks associated with point totals: $1-{{\hat {S}}_{0} \left( t \right) }^{\exp \left\{ {\mathop{\sum }\nolimits }_{i=1}^{p} \left( {\hat {\gamma }}_{i}\cdot {W}_{iREF} \right) +B\cdot Points-{\mathop{\sum }\nolimits }_{i=1}^{p}{\hat {\gamma }}_{i}\cdot \hat {{\bar {w}}_{i}} \right\} }$, where ${\hat {S}}_{0} \left( t \right) $ is calculated through the Kaplan–Meier estimator.

### Joint models for longitudinal and time-to-event data

Using the former notation, we have the random variables vector $ \left\{ T,W,Y \left( T \right) \right\} $, where $Y \left( T \right) $ is only a time-dependent variable (longitudinal outcome) which has its values defined intermittently for *t*. In other words, for a subject $ \left( i=1,\ldots ,n \right) $, *y*(*t*) is only defined for ${t}_{ij} \left( j=1,\ldots ,{n}_{i} \right) $, ${y}_{i} \left( {t}_{ij} \right) $, where 0 ≤ *t*_*i*1_ ≤ *t*_*i*2_ ≤ … ≤ *t*_*in*_*i*__. Now, we will denote as $m \left( t \right) $ the true and unobserved value of the longitudinal outcome at time *t* (${m}_{i} \left( t \right) $ for the subject *i*). To assess the effect of $m \left( t \right) $ on the event risk, a standard option is to adjust a Cox regression model with one time-dependent covariate:

\begin{eqnarray*}h \left( t{|}M \left( t \right) ,w \right) ={h}_{0} \left( {t}^{\ast } \right) \exp \left\{ {\gamma }^{T}w+\alpha m \left( t \right) \right\} , \end{eqnarray*}

where $M \left( t \right) $ for a subject *i* is defined as ${M}_{i} \left( t \right) = \left\{ {m}_{i} \left( u \right) ;0\leq u\lt t \right\} $, which denotes the history of the true unobserved longitudinal process up to time *t*. The other parameters in the expression follow the structure of the Cox regression model with time-dependent variables (see former section). The baseline risk function can be unspecified or can be approximated with splines or step functions (Rizopoulos, 2012).

In the above expression, we have used *m*(*t*) as the true unobserved longitudinal process. However, in our sample we have *y*(*t*); therefore, we will estimate *m*(*t*) using *y*(*t*) through a linear mixed effects model to describe the subject-specific longitudinal evolutions:

\begin{eqnarray*} \left\{ \begin{array}{@{}l@{}} \displaystyle {y}_{i} \left( t \right) ={m}_{i} \left( t \right) +{\varepsilon }_{i}(t)\\ \displaystyle \begin{array}{@{}l@{}} \displaystyle {m}_{i} \left( t \right) ={x}_{\mathbf{i}}^{T}(t)\beta +{z}_{\mathbf{i}}^{T}(t){b}_{\mathbf{i}}\\ \displaystyle {b}_{\mathbf{i}}\sim N \left( \mathbf{0},D \right) \\ \displaystyle {\varepsilon }_{i}(t)\sim N \left( 0,{\sigma }^{2} \right) \end{array} \end{array}, \right. \end{eqnarray*}

where *β* and *b*_**i**_ denote the vectors of regression coefficients for the unknown fixed-effects parameters and the random effects respectively, *x*_**i**_(*t*) and *z*_**i**_(*t*) denote row vectors of the design matrices for the fixed and random effects respectively, and ε_*i*_(*t*) is the error term with variance *σ*^2^. Finally, *b*_**i**_ follows a normal distribution with mean **0** and covariance matrix *D*, and independent of ε_*i*_(*t*) (Rizopoulos, 2012).

The estimation of the parameters of the joint models is based on a maximum likelihood approach that maximizes the log-likelihood function corresponding to the joint distribution of the time-to-event and longitudinal outcomes (Rizopoulos, 2012).

Regarding the assumptions of the model, we have to assess them for both submodels (longitudinal and survival) using the residual plots. For the longitudinal part, we will plot the subject-specific residuals versus the corresponding fitted values, the Q–Q plot of the subject-specific residuals, and the marginal residuals versus the fitted values. On the other hand, for the survival part, we will plot the subject-specific fitted values for the longitudinal outcome versus the martingale residuals, and finally we will determine graphically whether the Cox-Snell residuals is a censored sample from a unit exponential distribution (Rizopoulos, 2012). Regarding the last component (random effects part) of the joint model for which we have indicated an assumption, other authors have showed that linear mixed-effects models are relatively robust to misspecification of this distribution (Verbeke & Lesaffre, 1997).

### Predictions of the longitudinal biomarkers using these joint models for longitudinal and time-to-event data

Let $ \left\{ {t}_{i},{\delta }_{i},{w}_{i},{y}_{i} \left( {t}_{ij} \right) ,{0\leq t}_{ij}\leq {t}_{i},\hspace*{1em}j=1,\ldots ,{n}_{i} \right\} ,i=1,\ldots n$ be a random sample of the random variables vector {*T*, Δ, *W*, *Y*}, using the former notation. A joint model has been fitted using this sample. Now, we are interested in predicting the expected value of the longitudinal outcome at time *u* > *t* for a new subject *i* who has a history up to the time *t* of the observed longitudinal marker ${Y}_{i} \left( t \right) = \left\{ {y}_{i} \left( s \right) ;0\leq s\lt t \right\} $:

\begin{eqnarray*}{\omega }_{i} \left( u{|}t \right) ={E}_{Y} \left\{ {y}_{i} \left( u \right) {|}{t}_{i}^{\ast }\gt t,{Y}_{i} \left( t \right) ,{w}_{\mathbf{i}};\theta \right\} \end{eqnarray*}

where *θ* denotes the parameters’ vector of the joint model (Rizopoulos, 2011).

Rizopoulos developed a Monte Carlo approach to perform this task, based on Bayesian formulation. He obtained the following simulation scheme (Rizopoulos, 2011):

Step 1: Draw ${\theta }^{(l)}\sim N \left( \hat {\theta },\hat {var} \left( \hat {\theta } \right) \right) $.

Step 2 : Draw ${b}_{\mathbf{i}}^{(l)}\sim \left\{ {b}_{\mathbf{i}}{|}{t}_{i}^{\ast }\gt t,{Y}_{i} \left( t \right) ,{w}_{\mathbf{i}}\mathbf{;}{\theta }^{(l)} \right\} $.

Step 3: Compute ${\omega }_{i}^{(l)} \left( u{|}t \right) ={\mathbf{x}}_{\mathbf{i}}^{T} \left( u \right) {\beta }^{(l)}+{\mathbf{z}}_{\mathbf{i}}^{T} \left( u \right) {\mathbf{b}}_{\mathbf{i}}^{(l)}$.

This scheme should be repeated *L* times. The estimation of the parameter is the mean (or median) of the calculated values (${\omega }_{i}^{(l)} \left( u{|}t \right) ,l=1,\ldots L$) and the confidence interval is formed by the percentiles (95%: 2.5% and 97.5% percentiles) (Rizopoulos, 2011).

We highlight that these predictions have a dynamic nature; that is, as time progresses additional information is recorded for the patient, so the predictions can be updated using this new information.

### Construction

We wish to determine the probability of having CVD with effect from a baseline situation (*t* = 0) up to a fixed point in time ($\tilde {t}$), given a series of risk factors measured at baseline and during this follow-up. To do this requires the following steps:

(1) Adjust a Cox regression model with time-dependent variables. As we are unable to estimate a joint model with multiple longitudinal parameters (Rizopoulos, 2012), we use the classic extended Cox model (with no shared structure), which requires knowing the values of all the longitudinal parameters at any value of *t*. As this is not known because the parameters are recorded intermittently, we take the last value in time as a reference.
(2) Use the procedure of the Framingham study to adapt the coefficients of the model obtained to a points system and determine the probabilities of CVD for each score up to the moment $\tilde {t}$. We then use these probabilities to construct risk groups that are easy for the clinician to understand (for example, in multiples of 5%) (Sullivan, Massaro & D’Agostino, 2004).
(3) Adjust a joint model for longitudinal and time-to-event data for each longitudinal parameter recorded during the follow-up. This will also include all the baseline variables. These models are constructed to make predictions about the longitudinal parameters in new patients (statistical validation by simulation and potential utilization).

### Statistical validation by simulation

Once the points system has been constructed, we wish to see whether the model determines the onset of CVD accurately in a different set of subjects (validation sample). In this validation sample we know the longitudinal markers up to the point *t* = 0 (record of cardiovascular risk factors in the clinical history which were measured before the baseline situation (*t* < 0)) and the value of the variables at baseline. With this information we determine the probability each subject has of experiencing an event, and we then compare this with what actually occurred; i.e., determine whether the model is valid. To determine this validity we follow these steps:

(1) Determine *L* simulations of the longitudinal parameters at the time point $\tilde {t}$ using the models mentioned in step (3) of construction, from the history (*t* < 0) and the baseline variables (*t* = 0) (Rizopoulos, 2011). We will use these simulated values to construct a distribution of the points for each sample subject. Thus, each subject will have *L* values for the points variable (evaluating the points system using the simulated values and the baseline variables is sufficient), and for each *l*th simulation each patient will have a points score. In other words, each simulation will have a distribution of the points variable.
(2) For each *l*th simulation adjust a classic Cox model (without time-dependent variables), using just the score obtained as the only explanatory variable. Determine the Harrell’s concordance statistic for each of these *L* models. These values will give us a distribution of values for this statistic, with which we calculate the mean (or the median) and the 2.5% and 97.5% percentiles (Rizopoulos, 2011). This way we construct a confidence interval for this statistic, which will indicate the discriminating capacity of the points system to determine which patients will develop CVD.
(3) Calculate the median of the points distribution for each patient in the validation sample. Note that we do not use the mean as it could contain decimals and this has no sense when applying the scoring system. Using these medians, classify each patient in a risk group and compare the rate of events predicted by the points system in each group to the actual observed rate. The test used for this process will be Pearson *χ*^2^ test.

The concordance statistic used has been reported to have various limitations (Lloyd-Jones, 2010). For example, it does not compare whether the estimated and observed risks are similar in the subjects. Accordingly, we have added the analysis of the differences between the expected events and the observed events, which minimises this particular problem. In addition, it is very sensitive to large hazard ratio values (≥9). Nonetheless, we have to consider that as all the variables are quantitative (not categorized), the hazard ratio values do not surpass this threshold. Accordingly, the joint analysis of the concordance index of Harrell and the differences between the expected and the observed events enables us to validate statistically by simulation of the proposed model.

### Explanation of potential utilization

Once the points system has been validated statistically the clinician can then apply the system to determine the cardiovascular risk in a new patient, and take any necessary measures to reduce this risk. The healthcare professional will already have historical information about the longitudinal parameters (*t* < 0) and information about the baseline situation (*t* = 0) of the new patient. The steps to be followed by the clinician are:

(1) Determine the value of each longitudinal parameter at the time $\tilde {t}$. To do this we apply the models obtained in step (3) of construction to the history and the baseline situation of the new patient, in order to determine *L* simulations for each longitudinal parameter, similar to what was done in the validation process. For each *l*th simulation we determine the score corresponding to the profile of cardiovascular risk factors obtained (simulated and baseline information values). This will give us a points distribution for the new patient.
(2) Determine the median and the 2.5% and 97.5% percentiles of the points vector constructed above. The median will be the estimation of the score for the new patient and the percentiles will define the confidence interval (Rizopoulos, 2011). As each score has an associated risk, the healthcare professional will be able to know the probability of CVD at time $\tilde {t}$, together with its confidence interval. Finally, the clinician will know the values of the biological parameters at $\tilde {t}$ of the median of the points system. This way the clinician will be able to see which of these parameters has a score above normal; i.e., see the possible areas of intervention to reduce the cardiovascular risk.
(3) The clinician now knows the cardiovascular risk and which parameters have a score above normal, so he or she can then design the best intervention for that patient. This presents a problem, as we need to know the value of each biological parameter at time $\tilde {t}$; i.e., the clinician knows an approximation based on simulations constructed from the patient history but does not know how the interventions will affect the cardiovascular risk.

From the previous step the clinician knows the parameters on which to act and the history of these parameters as well as the baseline situation. From these measurements the clinician can establish a realistic objective for the next patient visit at time $\tilde {\tilde {t}}(0\lt \tilde {\tilde {t}}\lt \tilde {t})$. The clinician now inserts the desired value of the biological parameter at $\tilde {\tilde {t}}$ and determines its value at time $\tilde {t}$; i.e., determine *L* simulations for each cardiovascular risk factor using the previous models (step 3 of construction), adding a new value to the history ($\tilde {\tilde {t}}$).

These calculations will give the benefit of the intervention (estimation (mean or median) of the biological parameter at $\tilde {t}$) and the clinician will be able to see from the points system how the patient’s risk will be reduced.

### Simulation on a data set

With the sole purpose of explaining how to use the method proposed here, we have simulated a data set upon which to apply each of the steps described above. Note that we are in fact going to simulate two data sets, one to construct the model and the other to validate it statistically via simulation. So that both sets are biologically plausible we have used estimations obtained in the Puras-GEVA cardiovascular study, which has been published in Medicine (Artigao-Ródenas et al., 2015).

Our data sets will include the following biological parameters: age (years), systolic blood pressure (SBP) (mmHg), HbA1c (%), atherogenic index, gender (male or female) and smoking (yes or no). Of these, the SBP, HbA1c and the atherogenic index will be present at baseline (*t* = 0) and in the follow-up for the construction sample (*t* > 0) or recorded in the clinical history for the statistical validation sample via simulation (*t* < 0). The choice to include these variables was based on the current cardiovascular risk scales (Conroy et al., 2003; National Heart, Lung, and Blood Institute, 2015), except for HbA1c, which is used instead of a diagnosis of diabetes mellitus in order to include another time-dependent parameter in the final model, in addition to which this way enables us to value the control of the diabetes mellitus (HbA1c < 6.5%) when preventing CVD.

For the main variable (time-to-CVD) we shall suppose that our cohort is used to predict CVD with a follow-up of 2 years. Note that the traditional cardiovascular risk scales use a time of 10 years (Conroy et al., 2003; National Heart, Lung, and Blood Institute, 2015). We have used this lower value because we are going to make predictions for the longitudinal parameters with effect from the baseline situation (*t* = 0) up to the prediction time and if we take a prediction value of 10 years the predictions for the longitudinal parameters will vary greatly and not allow us to make precise predictions about which patient will develop CVD, which would negate the usefulness of the method proposed here. Nevertheless, the fact that the predictions for the longitudinal parameters have a dynamic character (see *Predictions of the longitudinal biomarkers using these joint models for longitudinal and time-to-event data*) enables us to determine the risk at 2 years with greater precision whenever the patient attends the office of the healthcare professional. Note that the method proposed here has been developed for a theoretical time period $\tilde {t}$ but it can be applied for any time period. Nonetheless, generally speaking the longitudinal parameters would vary more over longer time periods, though this clearly depends on the nature of the data, both at the individual level and the population level (Rizopoulos, 2012).

The work used for our simulated data set developed and validated a predictive model of CVD (angina of any kind, myocardial infarction, stroke, peripheral arterial disease of the lower limbs, or death from CVD), to enable calculation of risk in the short, medium and long term (the risk associated with each score was calculated every 2 years up to a maximum of 14) in the general population (Artigao-Ródenas et al., 2015). Table 4 of this scoring system shows the importance of this question. For example, a patient with a score of 9 points has a probability of CVD at 2 years of 0.67%, whereas at 10 years this rises to 5.16% (Artigao-Ródenas et al., 2015: Table 4). If we regularly calculate the 2-year risk of CVD for our patient and the score remains the same then no new therapeutic action will be taken (risk < 1%), whereas if we only calculate the risk once every 10 years we will take aggressive therapeutic measures when the patient first attends the office, as the score will correspond to a cut point defined as high in the SCORE project (5% → one in 20 patients) (Conroy et al., 2003). We see, then, that a regular short-term prediction could lead to a change in the therapeutic decisions regarding prevention of CVD, provided of course that the possibility exists of calculating the risk regularly. As the risk table given in the Puras-GEVA study includes predictions for 4, 6, 8, 10, 12 and 14 years, we selected the lowest cut point because if we had to make predictions for a longer time the dispersion could have increased (Rizopoulos, 2012). This is why we chose this cut point of 2 years for the simulation.

The longitudinal follow-up measurements (construction sample) assumed that the patient attends the physician’s office once every 3 months for measurements of SBP, HbA1c and the atherogenic index. This is done until the end of the follow-up for each patient. The statistical validation sample using simulation supposes that there is a certain probability of having records in the clinical history of all the longitudinal parameters every 3 months for 5 years retrospectively (*t* < 0). The probability is different for each of the visits and will depend on each patient. In other words, we will have intermittent measurements of all these parameters from *t* = − 5 years to *t* = 0.

The Supplemental Information (S1) details all the mathematical formulae used to construct our data sets, always based on the Puras-GEVA study (Artigao-Ródenas et al., 2015). The simulation was done using R 2.13.2 and IBMS SPSS Statistics 19.

One could think that by managing a shorter time period of just 2 years there would be no variability in the cardiovascular risk factors. However, in S1 we can see that the models used show a temporal variability in the risk factors. If there were no variability in the factors, the models would contain the constant with a very small random error. In other words using this prediction time makes sense.

We decided to use a simulated data set as we did not have available any data set with real data. This way of explaining a new method has already been used by others working with joint models, as the only objective of the simulated data set is to explain how to apply the new method (Faucett & Thomas, 1996; Henderson, Diggle & Dobson, 2000; Wang & Taylor, 2001; Brown, Ibrahim & DeGruttola, 2005; Zeng & Cai, 2005; Vonesh, Greene & Schluchter, 2006; Rizopoulos & Ghosh, 2011).

## Results

Given the amount and extension of the results these are given in detail in the Supplemental Information (S2 and S3). However, we have provided here the main results of our example. As before, the analysis was done with R 2.13.2 and IBM SPSS Statistics 19.

### Construction of the model

**Figure 1:**
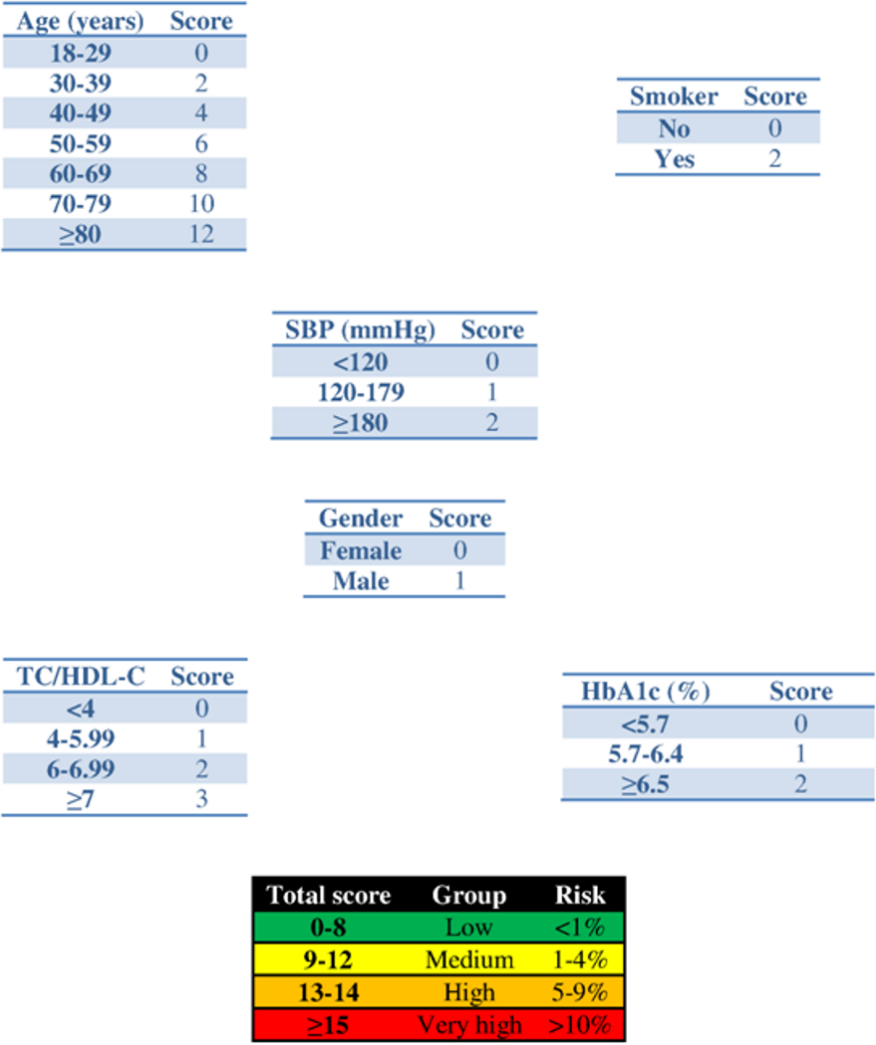
Scoring system to predict cardiovascular diseases within 2 years. Abbreviations: SBP, systolic blood pressure; HbA1c, glycated haemoglobin; TC, total cholesterol; HDL-c, HDL cholesterol.

The parameters of the Cox model with time-dependent variables are shown in Table 1, and its adaptation to the points system with a prediction time of 2 years is reflected in Fig. 1. Table 2 shows the joint models for the longitudinal parameters. To avoid computational cost simple models were used: (1) linear equation for the survival part with all the predictors included (age, gender, smoking, and longitudinal marker) and (2) mixed linear model with polynomial degree 1 at (1, *t*), in both the fixed and the random parts. The baseline risk function was defined piecewise.

**Table 1:** Parameters (*β* s) of the multivariate Cox regression model. Goodness-of-fit (likelihood ratio test): *χ*^2^ = 912.3, *p* < 0.001.

Variable	*β*	*p*-value
Age (baseline) (per 1 year)	0.0846	<0.001
SBP (per 1 mmHg)	0.00874	<0.001
HbA1c (per 1%)	0.188	<0.001
Atherogenic index (per 1 unit)	0.191	<0.001
Male gender	0.479	0.001
Smoker (baseline)	0.721	<0.001

**Notes.**

Abbreviations SBP Systolic blood pressure HbA1c glycated haemoglobin

**Table 2:** Parameters of the joint models with the longitudinal parameters studied. The strategy to eliminate variables is to eliminate down from the most complex terms to the most simple terms. Goodness-of-fit: (1) SBP: *χ*^2^ = 371, 574.1, *p* < 0.001; (2) HbA1c: *χ*^2^ = 210, 881.1, *p* < 0.001; (3) Atherogenic index: *χ*^2^ = 121, 118.0, *p* < 0.001.

Variable	SBP (mmHg)	*p*-value	HbA1c (%)	*p*-value	Atherogenic index	*p*-value
**Event process**						
Male gender	0.428	<0.001	0.475	<0.001	0.446	<0.001
Age (per 1 year)	0.0837	<0.001	0.0840	<0.001	0.0833	<0.001
Smoker	0.731	<0.001	0.757	<0.001	0.775	<0.001
Parameter (per 1 unit)	0.0085	<0.001	0.216	<0.001	0.195	<0.001
**Longitudinal process: fixed effects**						
1	133.557	<0.001	6.158	<0.001	4.602	<0.001
*t*	0.0046	<0.001	0.0001	<0.001	0.0001	<0.001
**Longitudinal process: random effects**						
1	21.683	N/A	1.346	N/A	1.324	N/A
*t*	0.0358	N/A	∗	∗	0.0013	N/A
Residual	8.933	N/A	0.357	N/A	0.302	N/A

**Notes.**

Abbreviations SBP systolic blood pressure HbA1c glycated haemoglobin N/A not applicable * term eliminated due to convergence problems

### Statistical validation by simulation

**Figure 2:**
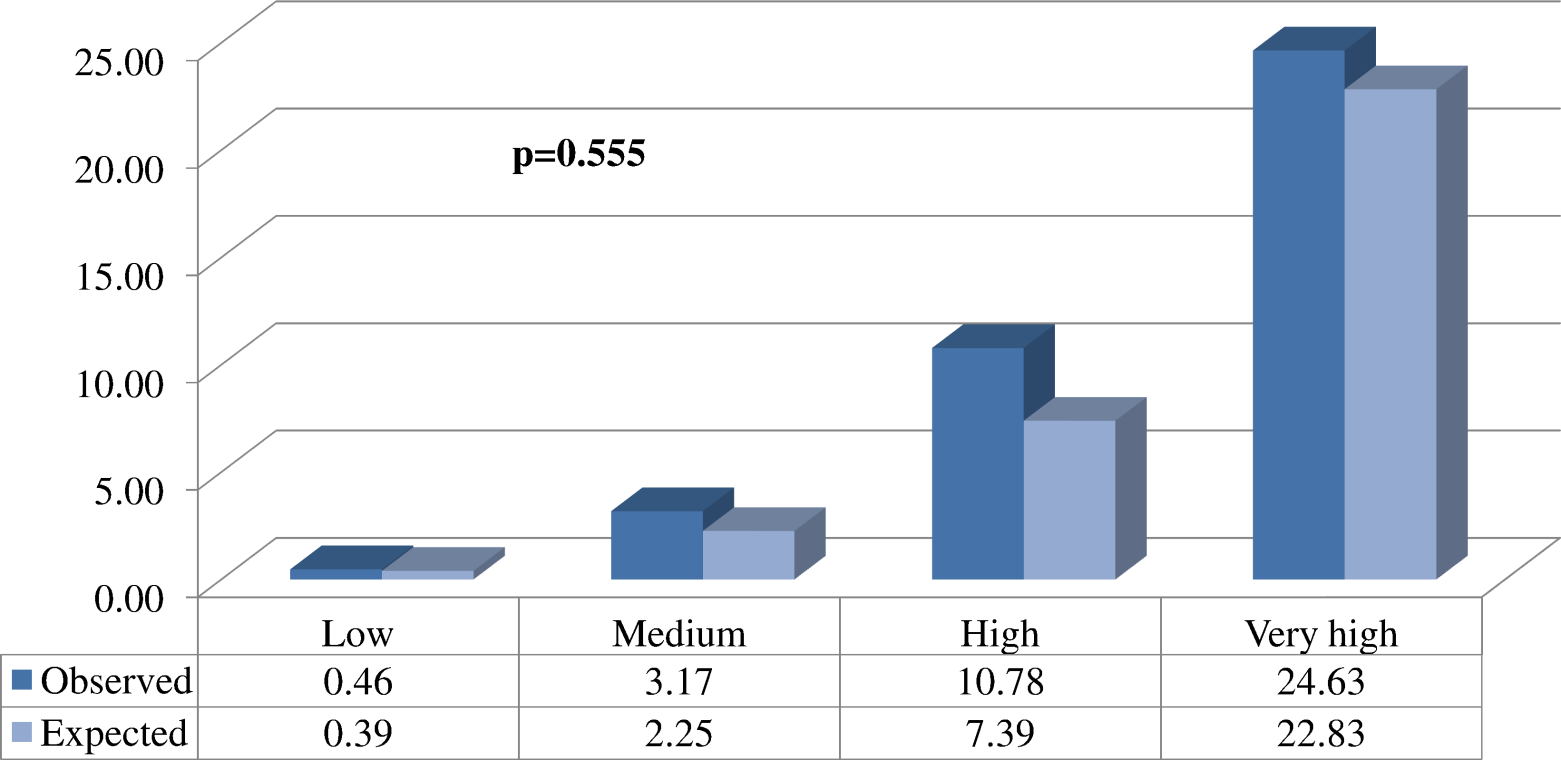
Comparison between the proportions (%) of expected and observed events in each of the different risk groups.

The *C*-statistic was very satisfactory: 0.844 (95% CI [0.842–0.846]). Comparison between expected and observed events in all the risk groups showed no significant differences (Fig. 2).

### Explanation of potential utilization

A new patient arrives at our office with the following characteristics: male, 83 years old, non-smoker, and taking pharmacological medication (one antihypertensive drug and one oral antidiabetic agent) and non-pharmacological measures (diet and exercise). His history of cardiovascular risk factors is available (Table 3).

**Table 3:** History of the control parameters of the cardiovascular risk factors included in our points system. Time has a negative value because it refers to the measurements taken before the baseline situation and this was defined as *t* = 0.

Time (days)	SBP (mmHg)	HbA1c (%)	Atherogenic index
−360	152	5.1	3.56
−330	135	5.3	3.23
−270	164	4.7	3.45
−180	153	4.4	4.12
−90	170	5.0	4.15
0	145	4.9	5.17

**Notes.**

Abbreviations SBP systolic blood pressure HbA1c glycated haemoglobin

**Figure 3:**
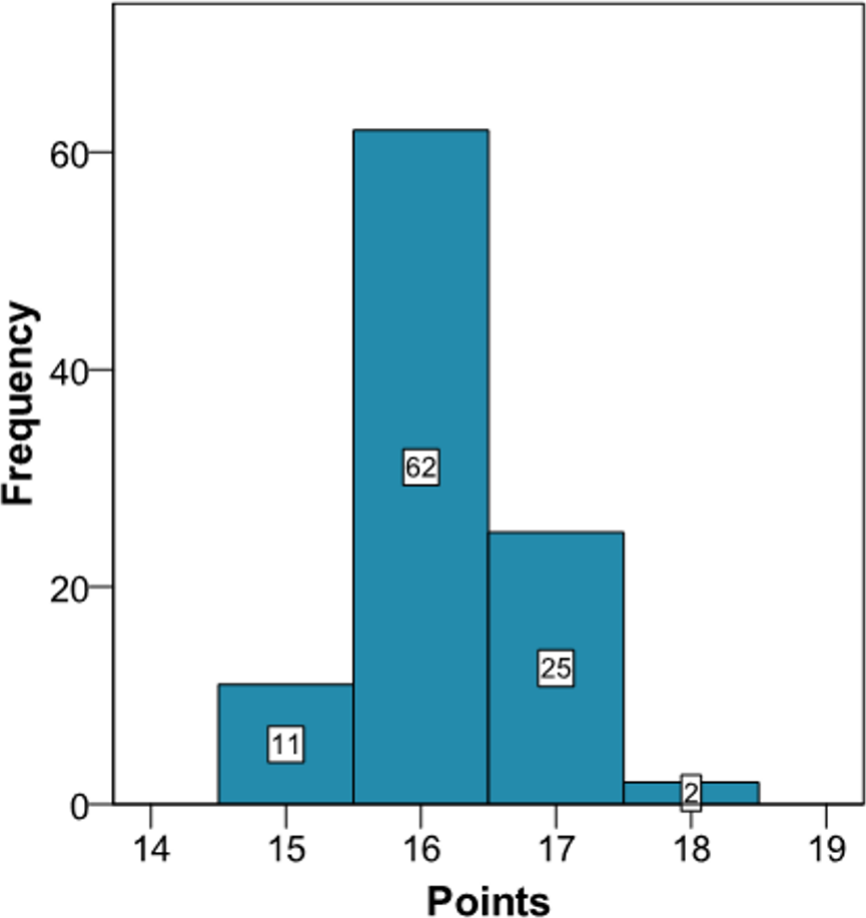
Cardiovascular risk of a theoretical new patient (pre-intervention).

Application of the new model gives a histogram of the cardiovascular risk score obtained for this patient (Fig. 3). This chart shows a high cardiovascular risk, as most of the simulations have around 16 points. The estimation of the score was 16 (95% CI [15–17]). The median score corresponded to a SBP of 160 mmHg, HbA1c of 5.0% and an atherogenic index of 6.76. Bearing in mind that the model contains factors upon which it is not possible to act (gender and age) that give the patient a minimum of 13 points, we should consider strategies to help the patient not to score in the other categories on the scale (Fig. 1).

**Figure 4:**
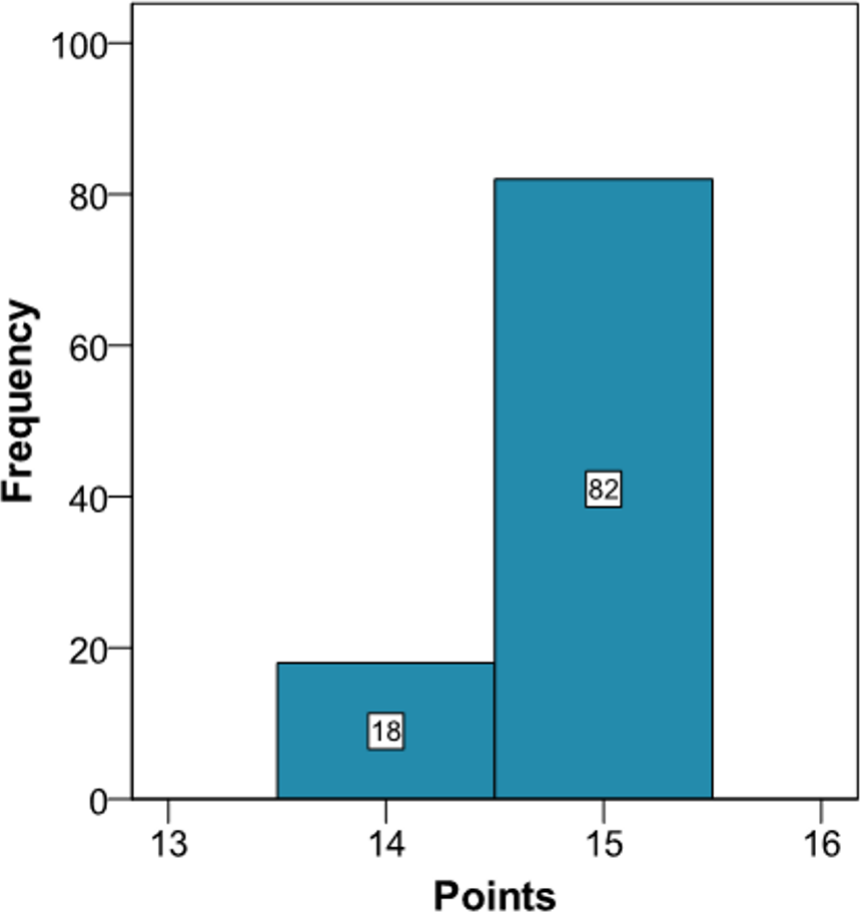
Cardiovascular risk of a theoretical new patient (post-intervention).

The clinician can now see that if the patient complies with a series of interventions (pharmacological (add two antihypertensive drug → −20 mmHg; prescribe a statin →−40% atherogenic index) and non-pharmacological (reduce salt in the diet →−5 mmHg)), his longitudinal parameters after 3 months would be: SBP 120 mmHg (145 – 2 × 10 – 5 = 120 mmHg), atherogenic index 3.10 (5.17 – 40% = 3.10), and HbA1c 4.9% (same value because no intervention was done). Applying the model using the new information gives the cardiovascular risk at 2 years (Fig. 4). The estimation of the score is 15 (95% CI [14–15]) and the values that provide a median score are: SBP 124 mmHg, atherogenic index 4.85, and HbA1c 5.0%. Thus, the risk is reduced, as now the patient has 15 points (Fig. 1 and S3).

## Discussion

This paper describes a method to construct predictive models for CVD considering the variability of cardiovascular risk factors and at the same time having the simplicity of points systems, which are widely used in daily clinical practice worldwide (Conroy et al., 2003; Cooney, Dudina & Graham, 2009; National Heart, Lung, and Blood Institute, 2015).

The cardiovascular risk scales currently available do not value the temporal variability of the parameters controlling the risk factors, although a very positive aspect of these scales is that they take into account simplicity for immediate application by healthcare professionals, the persons who really have to apply these mathematical models (Conroy et al., 2003; Cooney, Dudina & Graham, 2009; National Heart, Lung, and Blood Institute, 2015). The joint models currently used do take into account variability over time of a single longitudinal parameter (Rizopoulos, 2011), but their interpretation is not as easy as a points system and they cannot be used with various longitudinal parameters, a key question in the multifactorial aetiology of CVD. We have attempted to fuse all these techniques into one single algorithm, retaining the virtues of each (relative risks model, scoring systems, dynamic predictions...).

Comparison between our proposed model and current cardiovascular risk scales is problematic. Our model is more suitable to make short-term predictions, though the more time that passes from the baseline situation (*t* = 0) when making a prediction, the variability of the predictions of the longitudinal parameters increases (Rizopoulos, 2012). This same situation can be found in other areas, such as the economy (stock exchange) or meteorology (weather forecast), though it obviously depends on the nature of the data being used, both at the individual level and the population level. This however does not weaken our model, since because the predictions for the longitudinal parameters are dynamic (Rizopoulos, 2011); any time that we update the clinical information about our patient the risk is immediately recalculated. This can be seen in the proposed example (S2 and Figs. 3 and 4), where when we introduce new values for the longitudinal parameters these are updated and a new score for the patient is calculated. In other words, the proposed method could be used to calculate the patient’s risk every time the patient attends the office, whereas the traditional risk scales can be used with a longer time interval, as the prognosis is for 10 years. Thus, the two types of model could be used to assess the risk, for both the short term and the long term. Although discrepancies exist between short-term and long-term predictions of CVD (Quispe et al., 2015), the regular use of short-term predictions, bearing in mind the variability of the risk factors, can complement the long-term cardiovascular models. In other words, our intention is for clinical practice to use the short-term model regularly in those patients who attend their physician’s office frequently and use the long-term model in those who only attend occasionally.

Obtaining simulations from longitudinal parameters is not easy and implies a computational cost of about one minute with the statistical package R to implement a total of 100 using a normal computer. On the other hand, the historical values of the longitudinal parameters are recorded in the clinical history, which nowadays is usually electronic (Palazón-Bru et al., 2014). Given this situation, all the information needed to apply our models is already computerised, so the algorithms implemented in the statistical package R can be adapted to the underlying language of the database containing the values of the risk factors. Thus, all the calculations will be immediate for the healthcare professional. In other words, just pressing a key will be enough to bring up on the screen in a very short time the histogram shown in Fig. 2 and S2, the theoretical points system and the set of values of the risk factors determining the median score. In addition, when the physician decides to intervene he or she will indicate the duration of the intervention and the possible values for the new patient. After introducing this new information the two histograms could be shown together (Figs. 2 and 3, and S2), which will enable the physician to see the benefit of the intervention.

As this algorithm was developed from a set of simulated data, we encourage others who have cardiovascular databases like that used here to implement a model with the characteristics described herein. Thus, if using real-life data achieves greater predictive precision, we shall be able to apply this method to obtain the best short-term prognosis and thus take the most appropriate decisions for the benefit of the patient. Nevertheless, we should note that the method proposed is based on the combination of mathematical models already used in medicine; therefore, in theory our model is quite correct as we have been extremely strict in each of the steps to follow. In practice we can determine the value of $\tilde {t}$ and the complexity of the models in order to apply the method proposed. Finally, and importantly, the algorithm developed in this study can be used for other diseases or knowledge areas like the economy.

## Conclusions

We developed an algorithm to construct cardiovascular risk scales based on a points system that also takes into account the variability of the risk factors. These issues are important as the popularity of points systems in clinical practice and the improved predictive accuracy using all the information recorded in the clinical history will improve the currently used procedure. The theoretical construction of our method is based on the combination of mathematical models already used in medicine, taking into account the characteristics of each of these other models. As mentioned, the prediction time and the structure of each of the models can change in practice, as well as being used for other diseases apart from CVD or even applied to other areas of knowledge. Finally, as we do not have real data available for its immediate application in clinical practice, we encourage others to use our methods with their own data sets. In the case of CVD, traditional cohort studies should be done, but recording repeated measurements of risk factors both during the follow-up as well as for the period immediately prior to baseline

## Supplemental Information

10.7717/peerj.1673/supp-1 Supplemental Information 1 Construction of the data set

10.7717/peerj.1673/supp-2 Supplemental Information 2 Example given using simulated data

10.7717/peerj.1673/supp-3 Supplemental Information 3 Construction of the scoring system

## Additional Information and Declarations

### Data Availability

The following information was supplied regarding data availability: Our data set is simulated and we have explained how to obtain it in the Supplemental Information.
