# Supplementary material for: A method to construct a points system to predict cardiovascular disease considering repeated measures of risk factors
Source: PeerJ. 2016 Feb 15;4:e1673. doi: 10.7717/peerj.1673 (PMC4756731; doi:10.7717/peerj.1673)
Supplement: Supplemental Information 1 [file peerj-04-1673-s001.docx]

**CONSTRUCTION OF THE DATA SET**

Here we set out the mathematical formulae to construct the data sets:

1. We fix the total number of subjects for each set at 10,000.
2. Simulate baseline values ($t=0$) for each of the biological parameters to form part of the future scoring system, bearing in mind that each parameter will have a determined number of decimals and that it should also lie within a determined range of values. The distributions of each of these parameters can be seen in Table 1.

Table 1: Distributions of the simulated parameters for the baseline situation, according to data from the Puras-GEVA study.

| Variable | Formula | Decimal places | Range |
| --- | --- | --- | --- |
| Age (years) | $N\left( 47.42,{17.12}^{2} \right)$ | 0 | 18-89 |
| SBP (mmHg) | $N\left( 133.28,{21.46}^{2} \right)$ | 0 | 85-220 |
| HbA1c (%) | $N\left( 5.86,{1.58}^{2} \right)$ | 1 | 3.9-10.9 |
| Atherogenic index | $N\left( 4.51,{1.41}^{2} \right)$ | 2 | 1.92-10.84 |
| Male gender | $b(0.43)$ | 0 | 0-1 |
| Smoker | $b(0.31)$ | 0 | 0-1 |

Abbreviations: SBP, systolic blood pressure; HbA1c, glycated haemoglobin.

1. Simulate our main variable (time-to-cardiovascular disease):
   1. Determine the follow-up time (in days), which we have defined to have a distribution $N\left( 2*365,\left( \frac{365}{2} \right)^{2} \right)$, establishing a range from 90 days (so that each subject has at least 2 measurements in the construction sample) and 4 years (end of study).
   2. Constant baseline hazard per 1 unit time (1 day): 0.0000125.
   3. Using the following Cox regression model, $h_{0}\left( t \right)\exp\left\{ 0.100\cdot Age+0.03\cdot SBP+0.25\cdot HbA1c+0.35\cdot Atherogenic index+0.65\cdot male+0.70\cdot smoker \right\}$ [systolic blood pressure (SBP), glycated haemoglobin (HbA1c)], simulate the probability of event $p$.
   4. Simulate $u\sim Unif\left( 0,1 \right)$. If $u<p$ consider that the patient developed a cardiovascular disease; if not, consider no cardiovascular disease.
2. Simulate the repeated measures during the follow-up ($t>0$) for the patients in the construction sample. To do this we must consider that the data will be simulated for $t=90, 180, 270, \ldots, 1,440$ days until $t$ is greater than the follow-up time for each patient. For each parameter (SBP, HbA1c and atherogenic index) we shall determine the actual value at $t$ as the previous value plus a random quantity that will have two parts: the first part will depend on the variability of the biological parameter itself (inter) and the second part will depend on the variability of the parameter in each individual subject (intra). Table 2 shows the distributions used to obtain our simulations.

Table 2: Distributions of the simulated parameters for the follow-up, according to data from the Puras-GEVA study.

| Variable | Inter | Intra | Range |
| --- | --- | --- | --- |
| SBP (mmHg) | $N\left( 0,{2.5}^{2} \right)$ | $N\left( 0,\left( N\left( 10,1 \right) \right)^{2} \right)$ | 85-220 |
| HbA1c (%) | $N\left( 0,{0.1}^{2} \right)$ | $N\left( 0,\left( N\left( 0.25,0.1 \right) \right)^{2} \right)$ | 3.9-10.9 |
| Atherogenic index | $N\left( 0,{0.25}^{2} \right)$ | $N\left( 0,\left( N\left( 0.2,0.15 \right) \right)^{2} \right)$ | 1.92-10.84 |

This procedure is analogous in the statistical validation sample via simulation, except that the time will have a maximum of 5 years and the probability $\dot{p}$ that the patient will fail to attend the visit, so we will not have the relevant values recorded in the database. Nevertheless, the value will be calculated in order to obtain the next simulation value in time. The probability of having a measurement was considered with a distribution $N\left( 0.6,{0.1}^{2} \right)$ with a range $0-1$.
