# Supplementary material for: A method to construct a points system to predict cardiovascular disease considering repeated measures of risk factors
Source: PeerJ. 2016 Feb 15;4:e1673. doi: 10.7717/peerj.1673 (PMC4756731; doi:10.7717/peerj.1673)
Supplement: Supplemental Information 2 [file peerj-04-1673-s002.docx]

**CONSTRUCTION OF THE SCORING SYSTEM**

1) Estimate the multivariate Cox model with time-dependent variables defined as the last recorded value. The coefficients are shown in Table 1.

Table 1: Parameters (βs) of the multivariate Cox regression model.

| Variable | β | p-value |
| --- | --- | --- |
| Age (baseline) (per 1 year) | 0.0846 | <0.001 |
| SBP (per 1 mmHg) | 0.00874 | <0.001 |
| HbA1c (per 1%) | 0.188 | <0.001 |
| Atherogenic index (per 1 unit) | 0.191 | <0.001 |
| Male gender | 0.479 | 0.001 |
| Smoker (baseline) | 0.721 | <0.001 |

Abbreviations: SBP, systolic blood pressure; HbA1c, glycated haemoglobin.
Goodness-of-fit (likelihood ratio test): χ^2^=912.3, p<0.001.

The analysis of the Martingala residuals (Figure 1) shows that all the variables have a linear form in the model, as the red line has a perfect linearity. This is to be expected as the data were simulated this way. Nevertheless, it needs to be corroborated before continuing with the proposed model.

Figure 1: Functional form of the covariates in the model.

| 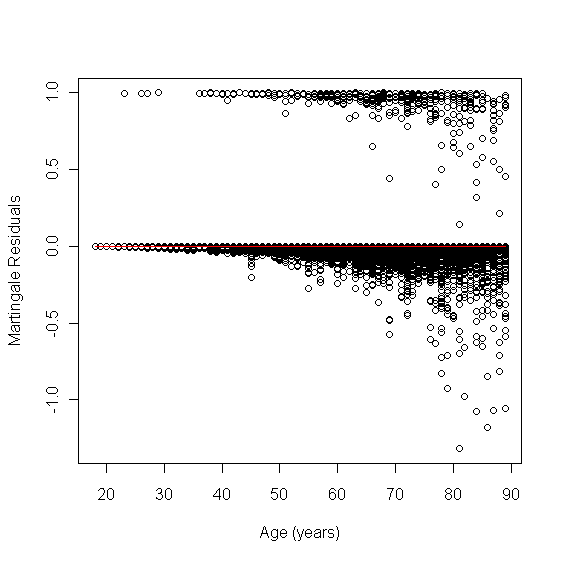 | 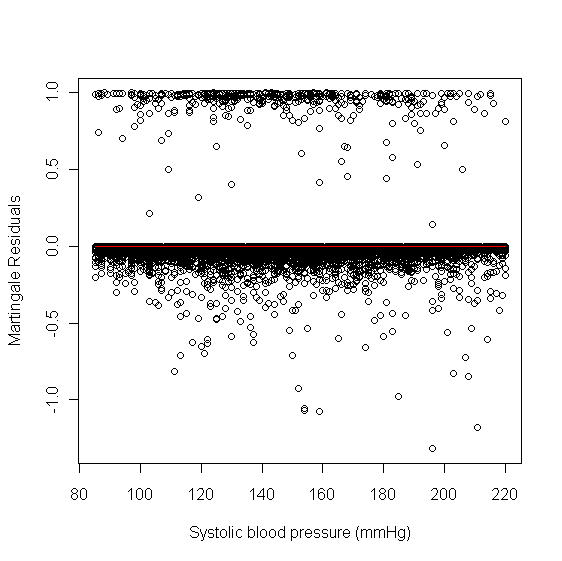 |
| --- | --- |
| 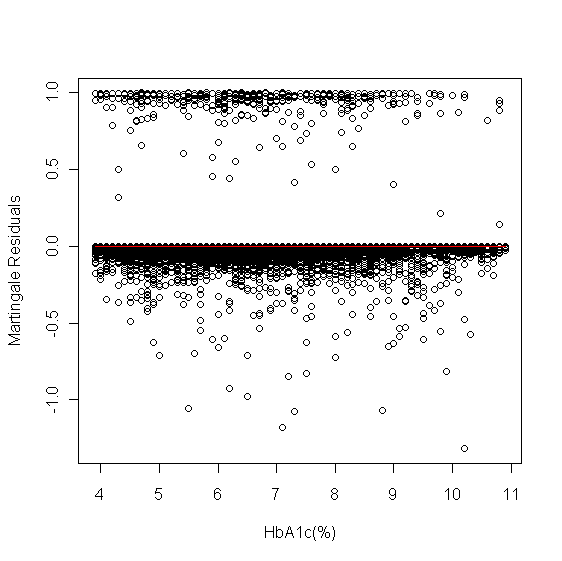 | 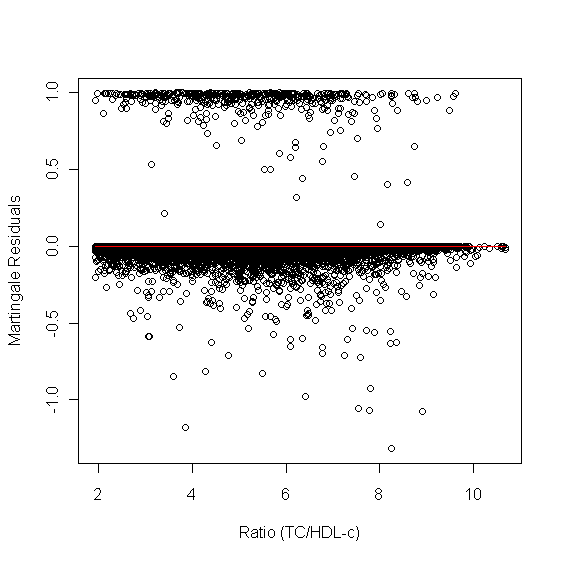 |
| 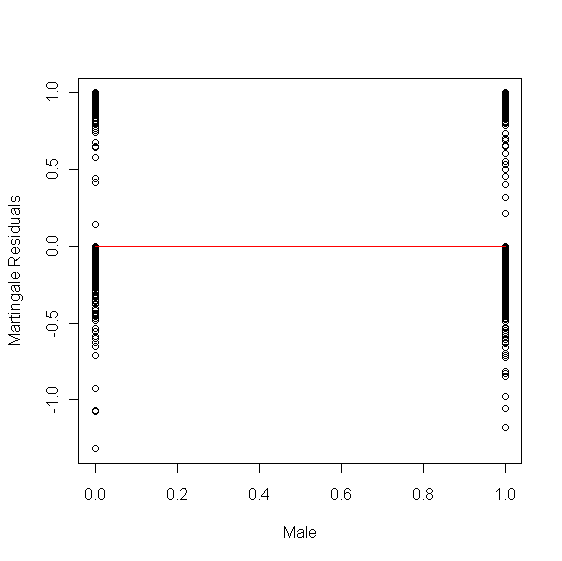 | 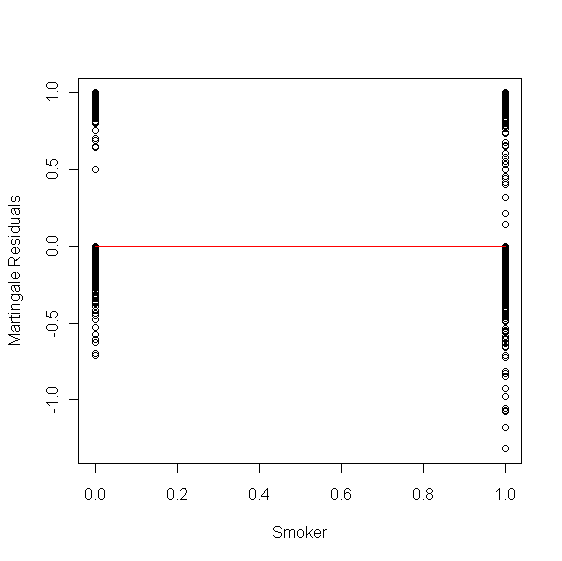 |

We then need to corroborate that the model adjusts the data well by the Cox-Snell analysis of residuals (Figure 2). As can be seen, the red line remains within the confidence intervals. Thus our model fulfils all the basic hypotheses and we can then start step 2) of the construction.

Figure 2: Analysis of the Cox-Snell residuals.


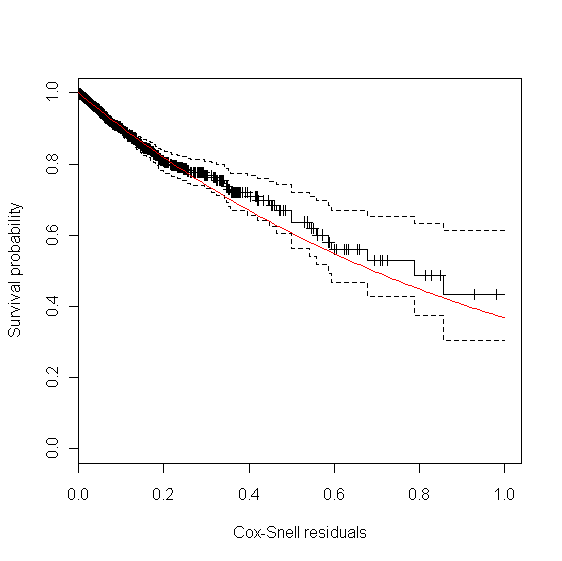


2) Adapt the coefficients obtained in the multivariate model to a points system using the procedure of the Framingham Heart Study. As this is a widely used procedure we just give the result in the form of a figure (Figure 3). Nonetheless, all the calculations are given in the annexed Excel spreadsheet in the supplementary material (Other S3). A figure was used rather than a table to aid comprehension, as the cells are separated more and there is less confusion for the clinician. This same idea has been used for both the SCORE project and the Framingham study. Note that the risk groups were constructed as for the SCORE project.

Figure 3: Scoring system to predict cardiovascular diseases within 2 years.


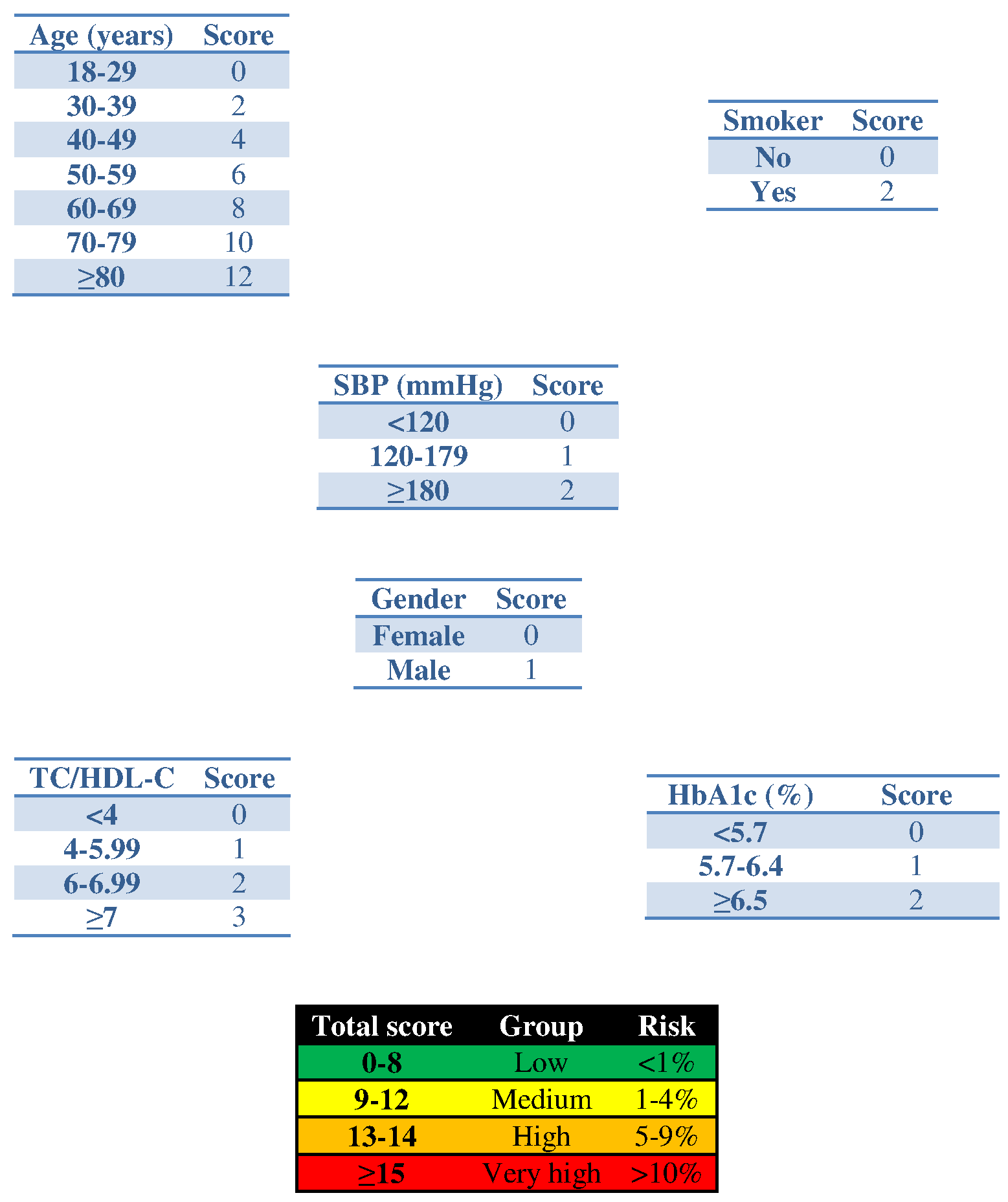


Abbreviations: SBP, systolic blood pressure; HbA1c, glycated haemoglobin; TC, total cholesterol; HDL-c, HDL cholesterol.

3) We have adjusted the joint models for each of the longitudinal parameters and their estimated coefficients can be seen in Table 2. No baseline risk function has been specified.

Table 2: Parameters of the joint models with the longitudinal parameters studied.

| Variable | SBP (mmHg) | p-value | HbA1c (%) | p-value | Atherogenic index | p-value |
| --- | --- | --- | --- | --- | --- | --- |
| Event process | | | | | | |
| Male gender | 0.428 | <0.001 | 0.475 | <0.001 | 0.446 | <0.001 |
| Age (per 1 year) | 0.0837 | <0.001 | 0.0840 | <0.001 | 0.0833 | <0.001 |
| Smoker | 0.731 | <0.001 | 0.757 | <0.001 | 0.775 | <0.001 |
| Parameter (per 1 unit) | 0.0085 | <0.001 | 0.216 | <0.001 | 0.195 | <0.001 |
| Longitudinal process: fixed effects | | | | | | |
| 1 | 133.557 | <0.001 | 6.158 | <0.001 | 4.602 | <0.001 |
| t | 0.0046 | <0.001 | 0.0001 | <0.001 | 0.0001 | <0.001 |
| Longitudinal process: random effects | | | | | | |
| 1 | 21.683 | N/A | 1.346 | N/A | 1.324 | N/A |
| t | 0.0358 | N/A | * | * | 0.0013 | N/A |
| Residual | 8.933 | N/A | 0.357 | N/A | 0.302 | N/A |

Abbreviations: SBP, systolic blood pressure; HbA1c, glycated haemoglobin; N/A, not applicable. *: term eliminated due to convergence problems. The strategy to eliminate variables is to eliminate down from the most complex terms to the most simple terms.
Goodness-of-fit: 1) SBP: χ^2^=371,574.1, p<0.001; 2) HbA1c: χ^2^=210,881.1, p<0.001; 3) Atherogenic index: χ^2^=121,118.0, p<0.001.

Concerning the basic hypotheses of the models, first we can see in Figure 4 that there is no tendency between the adjusted values and the specific residuals of each person, as the red line (linear adjustment between the two variables on the Cartesian axis) remains very near to $y=0$.

Figure 4: Charts of the specific residuals for each subject versus the values fitted to the model.

| 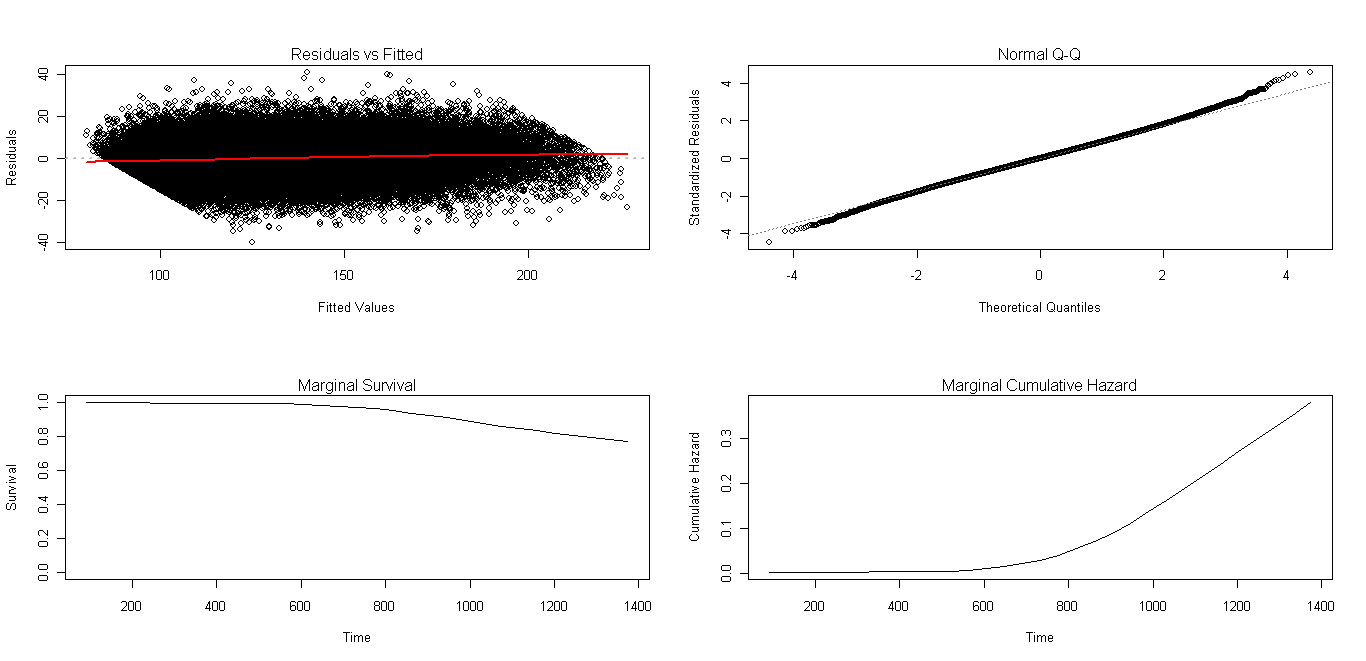 | |
| --- | --- |
| **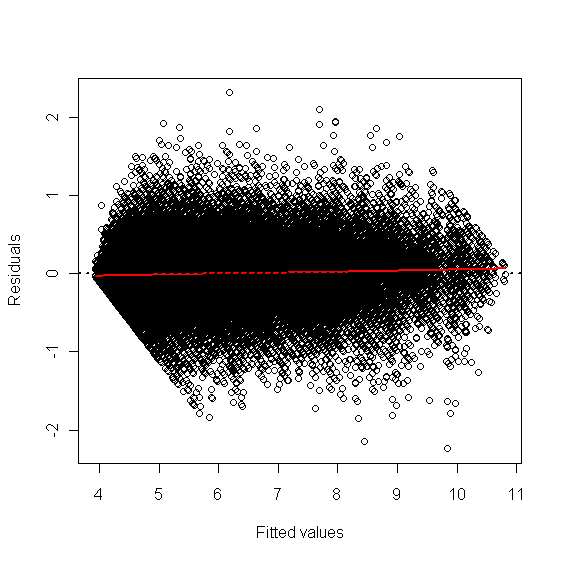** | **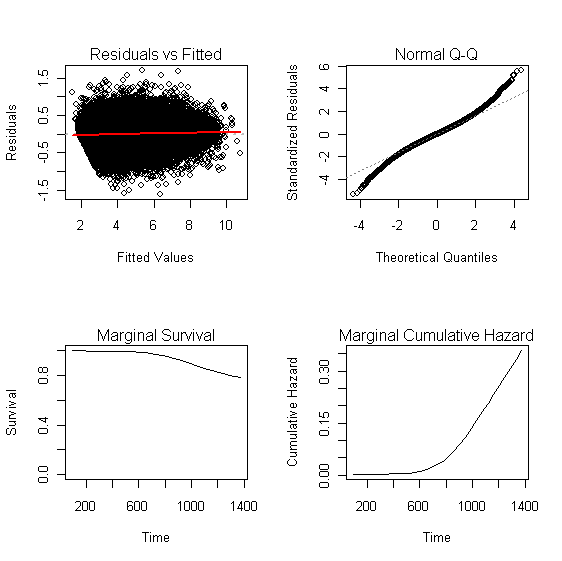** |

Top: systolic blood pressure. Bottom left: glycated haemoglobin. Bottom right: atherogenic index.

Concerning the normality of the specific residuals, given the amount of data available, we can assume asymptotic normality. Nevertheless, Figure 5 gives the Q-Q charts comparing the distribution of the data with a normal distribution. From these charts we can also conclude that our residuals follow a normal distribution.

Figure 5: Q-Q plots of the specific residuals for each subject.

| 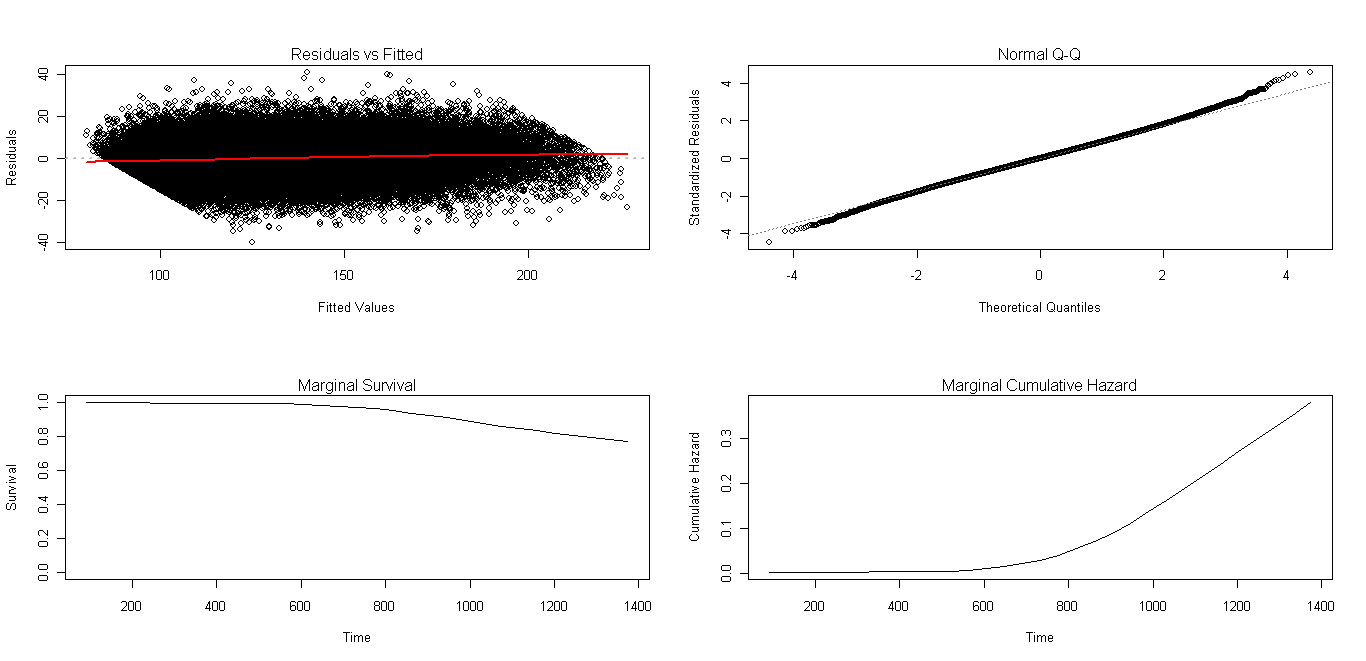 | |
| --- | --- |
| **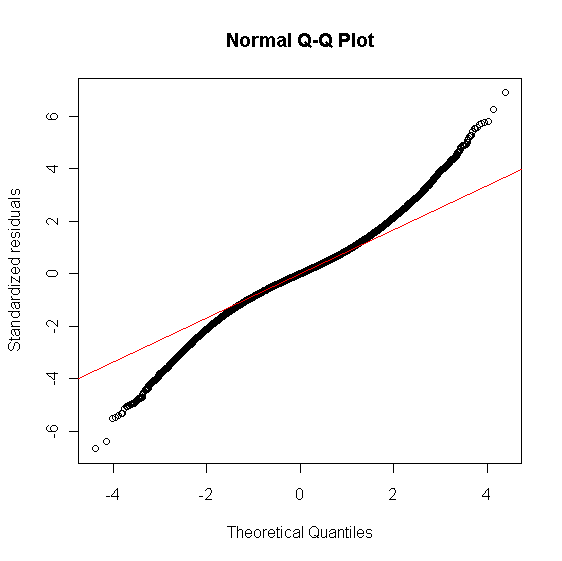** | **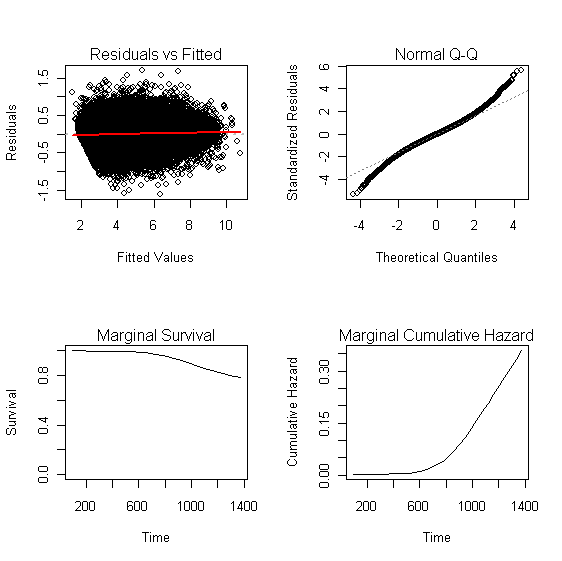** |

Top: systolic blood pressure. Bottom left: glycated haemoglobin. Bottom right: atherogenic index.

The analysis of the marginal residuals versus the adjusted values (Figure 6) shows that the red line (linear adjustment between the variables presented in the cartesian chart) established no pattern outside $y=0$. In other words, all the suppositions in the longitudinal submodel are verified. We can therefore check the basic hypotheses of the survival submodel.

Figure 6: Plots of the marginal residuals versus the adjusted values.

| **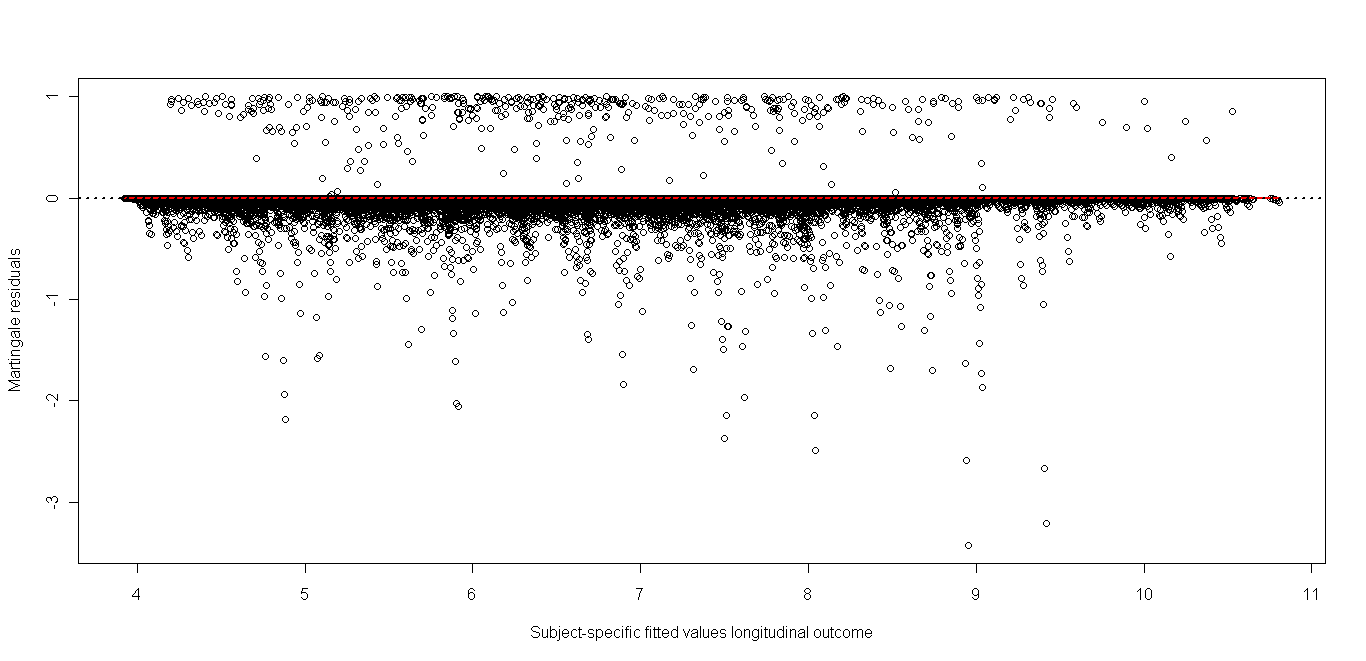** | |
| --- | --- |
| **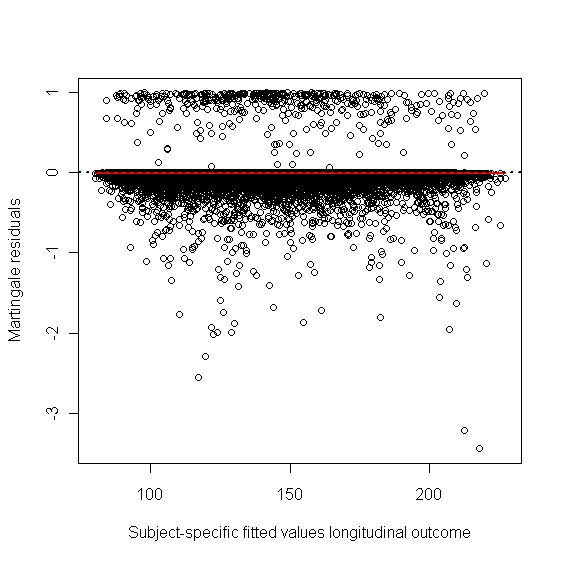** | **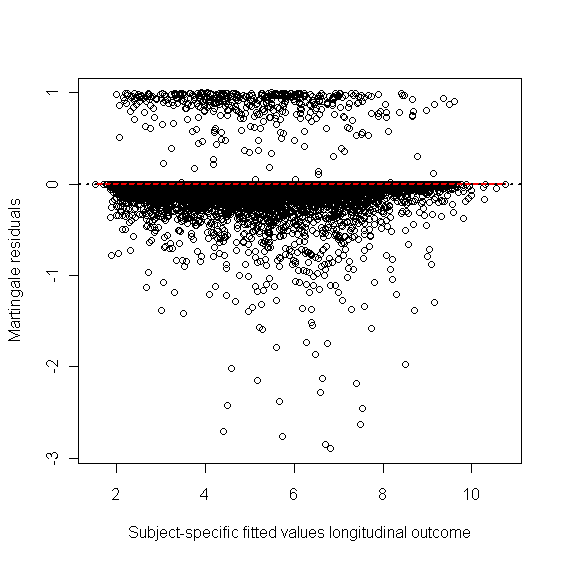** |

Top: glycated haemoglobin. Bottom left: systolic blood pressure. Bottom right: atherogenic index.

Figure 7 shows both types of charts to corroborate the basic hypotheses of the survival submodel. In the first column (adjusted values of the longitudinal parameters versus the Martingala residuals), there is no tendency, as the red line (linear adjustment between the two variables on the chart) resembles the line $y=0$. On the other hand we can assume that the Cox-Snell residuals follow an exponential distribution with unit mean (the red line lies within the survival confidence intervals).

Figure 7: Charts to corroborate the basic hypotheses of the survival submodel.

| **Martingala residuals** | **Cox-Snell residuals** |
| --- | --- |
| **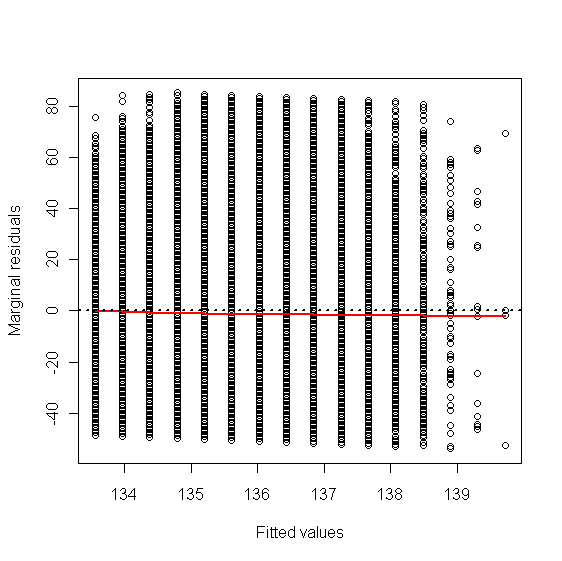** | **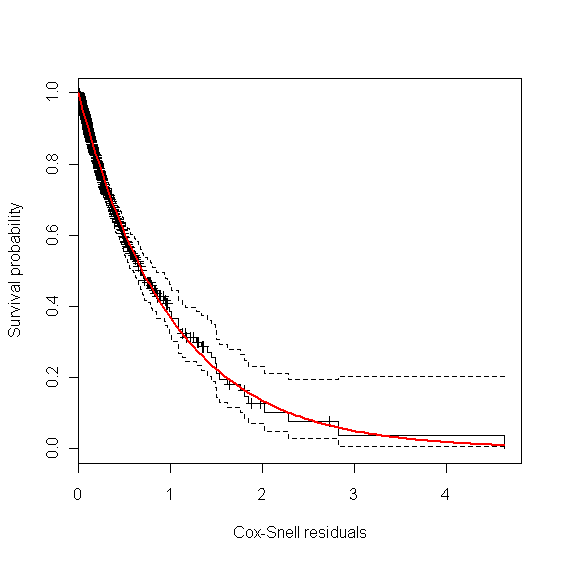** |
| **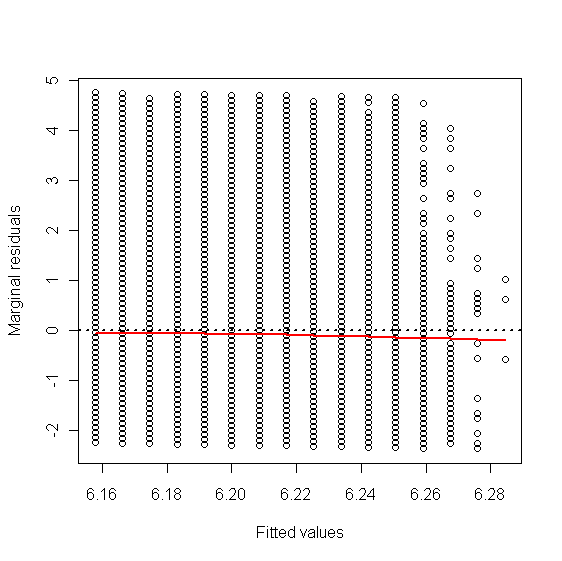** | **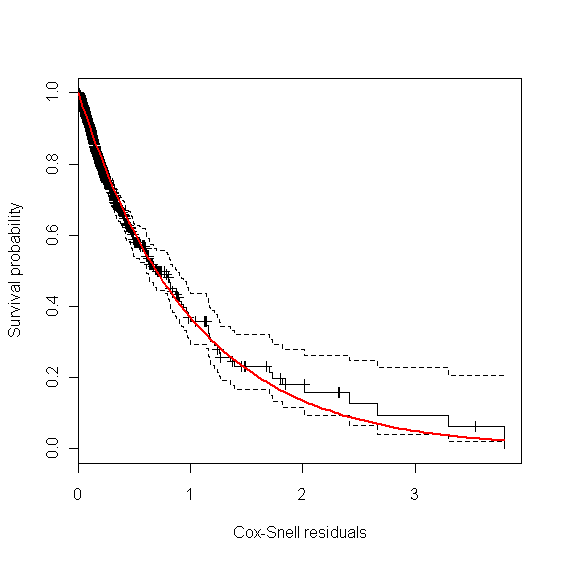** |
| **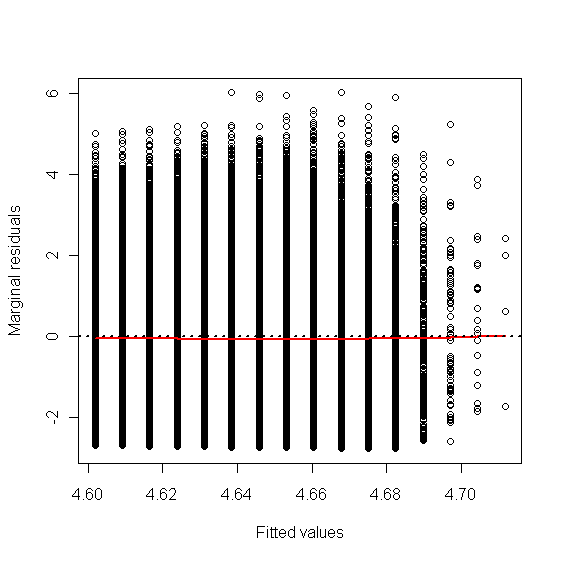** | **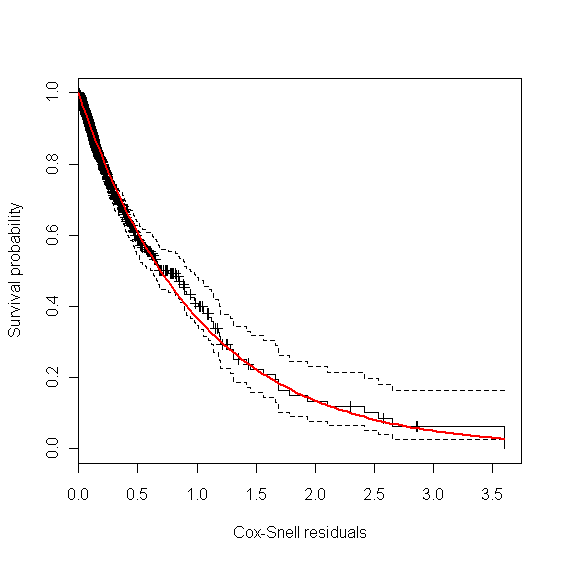** |

Top: systolic blood pressure. Middle: glycated haemoglobin. Bottom: atherogenic index.

**STATISTICAL VALIDATION BY SIMULATION OF THE SCORING SYSTEM**

The concordance was very satisfactory: 0.844 (95% CI: 0.842-0.846). Comparison between expected and observed events in all the risk groups showed no significant differences (Figure 8).

Figure 8: Comparison between the proportions (%) of expected and observed events in each of the different risk groups.

**EXPLANATION OF POTENTIAL UTILIZATION**

A new patient arrives at our office with the following characteristics: male, 83 years old, non-smoker, and taking pharmacological medication (one antihypertensive drug and one oral antidiabetic agent) and non-pharmacological measures (diet and exercise). His history of cardiovascular risk factors is available (Table 3).

Table 3: History of the control parameters of the cardiovascular risk factors included in our points system.

| Time (days) | SBP (mmHg) | HbA1c (%) | Atherogenic index |
| --- | --- | --- | --- |
| -360 | 152 | 5.1 | 3.56 |
| -330 | 135 | 5.3 | 3.23 |
| -270 | 164 | 4.7 | 3.45 |
| -180 | 153 | 4.4 | 4.12 |
| -90 | 170 | 5.0 | 4.15 |
| 0 | 145 | 4.9 | 5.17 |

Abbreviations: SBP, systolic blood pressure; HbA1c, glycated haemoglobin. Time has a negative value because it refers to the measurements taken before the baseline situation and this was defined as $t=0$.

Application of the new model gives a histogram of the cardiovascular risk score obtained for this patient (Figure 9). This chart shows a high cardiovascular risk, as most of the simulations have around 16 points. The estimation of the score was 16 (95% CI: 15-17). The median score corresponded to a SBP of 160 mmHg, HbA1c of 5.0% and an atherogenic index of 6.76. Bearing in mind that the model contains factors upon which it is not possible to act (gender and age) that give the patient a minimum of 13 points, we should consider strategies to help the patient not to score in the other categories on the scale (Figure 3).

Figure 9: Cardiovascular risk of a theoretical new patient (pre-intervention).

The clinician can now see that if the patient complies with a series of interventions [pharmacological (add two antihypertensive drug → -20 mmHg; prescribe a statin → -40% atherogenic index) and non-pharmacological (reduce salt in the diet → -5 mmHg)], his longitudinal parameters after 3 months would be: SBP 120 mmHg (145 – 2·10 – 5 = 120 mmHg), atherogenic index 3.10 (5.17 – 40% = 3.10), and HbA1c 4.9% (same value because no intervention was done). Applying the model using the new information gives the cardiovascular risk at 2 years (Figure 10). The estimation of the score is 15 (95% CI: 14-15) and the values that provide a median score are: SBP 124 mmHg, atherogenic index 4.85, and HbA1c 5.0%. Thus, the risk is reduced, as now the patient has 15 points (Figure 3 and S3 Other).

Figure 10: Cardiovascular risk of a theoretical new patient (post-intervention).
